# We Are Not Islands: The Role of Social Support in the Relationship between Perceived Stress during the COVID-19 Pandemic and Psychological Distress

**DOI:** 10.3390/ijerph20043179

**Published:** 2023-02-11

**Authors:** Anita Padmanabhanunni, Tyrone B. Pretorius, Serena Ann Isaacs

**Affiliations:** Department of Psychology, University of the Western Cape, Bellville 7530, South Africa

**Keywords:** anxiety, COVID-19, depression, hopelessness, mediation, moderation, perceived stress, social support, university students

## Abstract

COVID-19 containment measures, including social distancing, quarantine, and confinement, significantly impacted social connectedness and contributed to heightened levels of perceived stress. Prior research has established that protective factors can mitigate emotional distress. This study investigated the protective role of social support in the relationship between perceived stress and psychological distress among a sample of university students. Participants (*n* = 322) completed the Multidimensional Scale of Perceived Social Support, the Perceived Stress Scale, short forms of the Center for Epidemiological Studies Depression Scale, the trait scale of the State-Trait Anxiety Inventory, and the Beck Hopelessness Scale. The results indicated that high levels of perceived stress were associated with high levels of hopelessness, depression, and anxiety. In terms of direct and mediating effects, social support was significant for depression and hopelessness but not for anxiety. Furthermore, the relationship between perceived stress and depression was higher for those with high levels of social support than for those with lower levels of social support. The findings suggest that in addition to enhancing social support resources, interventions must assist students in managing the uncertainty and anxiety associated with the pandemic. Furthermore, students’ appraisals of support and the extent to which support is experienced as beneficial must also be examined prior to the implementation of interventions.

## 1. Introduction

The COVID-19 pandemic represented an unprecedented global public health emergency and precipitated heightened levels of fear and anxiety. To control the spread of the outbreak, governments across the world implemented disease containment measures, including lockdowns, social distancing protocols, work-from-home mandates, and quarantines [1]. Although effective in reducing the spread of the virus, these measures disrupted daily routines, interpersonal relationships, and social connectedness. In addition, the restrictions impacted economies and contributed to job losses and financial insecurity. For many people, the COVID-19 outbreak and measures to curb its spread were perceived as significant psychological stressors. Perceived stress is defined as the extent to which situations and events in one’s life are appraised as unpredictable, uncontrollable, and overwhelming [2]. The existing research has linked high levels of perceived stress to depression, anxiety, and post-traumatic stress disorder (e.g., [3]). Research conducted during the pandemic documented elevated levels of mental health disorders, potentially arising from the psychological stress associated with the outbreak. For example, in a systematic review and meta-analysis of longitudinal cohort studies examining changes in mental health prior to and during the pandemic, Robinson and colleagues [4] reported that compared to pre-pandemic levels, there was an overall increase in depression levels and mood symptoms. Similarly, in a systematic review and meta-analysis, Wu and colleagues [5] found increased levels of anxiety, depression, distress, and insomnia during the pandemic. Those with chronic diseases were at a higher risk of depression and anxiety, and frontline health care workers were reported to have elevated levels of insomnia. In a multicohort study of the general population undertaken across six European studies, Magnúsdóttir and colleagues [6] reported persistent elevated levels of anxiety and depression among those with severe COVID-19 symptoms. This was ascribed to worries about infecting others as well as concerns about the longer-term health implications of infection.

The existing research has highlighted that specific population groups are particularly at risk for developing negative mental health outcomes due to the differential impacts of the pandemic on these groups. University students represent one such group [7]. Pandemic containment measures meant the closure of institutions of higher learning and the transfer of learning and teaching to an online environment, which many students found difficult to engage with meaningfully [8]. This produced significant stress and student worries about academic performance and future career trajectory. Social distancing, lockdowns, and work-from-home mandates limited the opportunities available for establishing meaningful connections with others and reduced students’ access to social support [9]. Students also feared disease transmission and the possibility of their loved ones becoming infected. Excessive levels of stress represent a critical precipitating factor for adverse mental health outcomes [10,11]. A mental health survey of university students in the United Kingdom [7] reported high levels of anxiety and depression as well as low levels of resilience, all of which were attributed to pandemic restrictions and social isolation. Similarly, a longitudinal study of students in the United States [12] found greater symptoms of depression, anxiety, and stress, as well as the increased use of problematic coping strategies (e.g., alcohol and substance use). A cross-sectional study of students in India [13] reported heightened levels of fear of COVID-19, depression, and anxiety, while a Bangladeshi study [14] found that increased worries about COVID-19 were associated with greater depression and anxiety among university students.

Although pandemics are typically associated with increased levels of psychological distress, specific protective factors have been found to mitigate the relationship between stress and coping [15]. These protective factors include resilience, self-efficacy, active problem-solving, familial support, and optimism [16]. Research [17] conducted during previous pandemics (e.g., Ebola and SARS outbreaks) demonstrated that social support was a critical protective factor in reducing rates of mental health problems. However, COVID-19 containment measures such as self-isolation, social distancing, and quarantine protocols may have impacted individuals’ accessibility to social support networks. In addition, fear, anxiety, and confusion may influence the receptiveness and capacity of significant others to offer support [1]. The current study focused on the protective role of social support in the relationship between perceived stress and psychological distress during the COVID-19 pandemic.

Social support is categorized in terms of its function (e.g., emotional support, material support, and financial support) and by the type of provider (i.e., individual support provided by family, friends, and significant others or public support provided by the government and communities) [18]. For the purposes of this study, social support is broadly conceptualized as perceived and received social support [19]. While perceived social support refers to subjective appraisals of the availability, accessibility, and adequacy of social support networks, received social support entails the actual quantity of social support received [20]. Generally, perceived social support can come from various sources including friends, family, neighborhood- or community-level support, co-workers, and romantic partners. The existing research has established that the source of social support has an influence on mental health. For adolescents, for example, support from friends compared to family has been found to be beneficial in promoting mental health, whereas for elderly people, support from their spouse has a greater impact on well-being compared to support from their adult children [21]. Studies on university students (e.g., [22]) have reported that social support from family and friends has a substantial influence on emotional, social, and academic performance.

Existing research [19,20] has confirmed that perceived social support is more strongly related to mental health and well-being compared to the actual structure of social support networks and the quantity of support received. Qi and colleagues [23] reported a higher prevalence of mental health problems among Chinese adolescents with medium and low levels of perceived social support from significant others (e.g., family and friends). A study of Jordanian health care workers [24] reported that perceived social support was associated with lower levels of anxiety, depression, stress, and fear. In a study of the general population in Lebanon, Grey and colleagues [15] found that increased appraisals of social support were associated with a reduced risk of sleeping difficulties and depression. Social support is also a strong predictor of resilience and post-traumatic growth following disasters and exposure to traumatic events [25]. Government-provided support has also been found to be beneficial in promoting health. Ifdil and colleagues [26] highlighted the positive role of government-provided online services that offered reliable information about the pandemic and educated the public so as to potentially reduce stigma and anxiety. Similarly, studies from Malaysia [27] and Ireland [28] have underscored the substantial financial measures implemented by the government to reduce the economic impact of the pandemic and thereby promote stability. These types of supports can contribute to well-being among the general public. Neighborhood- or community-level support has also been identified as an important contributor to mental health and is defined as perceptions of one’s community as helpful, close, and trusting [29]. There is evidence [29] that community cohesion facilitates recovery efforts following large-scale disasters, promotes resilience, and buffers against adverse mental health outcomes. In sum, the existing literature has suggested that social support is essential for not only reducing negative mental health outcomes in the context of adversity but also promoting adaptation and coping.

The current study is grounded in Lazarus and Folkman’s transactional model of stress and coping [30], as well as the theoretical model of stress-buffering [31], which proposes that protective factors buffer or mitigate the negative associations between stressful life events and mental health. According to the transactional model [30], individual responses to stressful life events are influenced by a cognitive appraisal process. Once individuals are confronted with a stressor, they engage in both a primary (i.e., assessing the relevance of the stressor) and a secondary appraisal process (i.e., evaluating the resources available to cope with the stressor). The outcome of this appraisal process influences the coping responses selected and the extent to which individuals experience psychological distress. There has been considerable support for this theory [32]. Based on this model, the research question we investigated was whether social support mediates the relationship between perceived stress and psychological well-being.

## 2. Materials and Methods

### 2.1. Participants and Procedure

The participants were students (*n* = 322) at a university in the Western Cape province of South Africa. The registrar’s office at the university randomly sampled 1700 students. We constructed an electronic version of the instruments detailed below and emailed it to the students with an information sheet and an invitation to participate by completing the questionnaire. The data were collected during April–June 2022. We received responses from 322 students. A description of the sample is presented in Table 1.

Table 1 reveals that the sample mainly consisted of women (77%) who lived in an urban area (86.6%). The mean age of the sample was 26.01 (SD = 10.19). Eighty-six percent of the sample reported knowing people who had been infected with the coronavirus, 25.5% had tested positive for COVID-19 themselves, and 86.6% had received a COVID-19 vaccine.

### 2.2. Measures

Participants completed the Multidimensional Scale of Perceived Social Support (MSPSS) [33], the Perceived Stress Scale (PSS) [2], as well as short forms of the Center for Epidemiological Studies Depression Scale (CESD-10) [34], the trait scale of the State-Trait Anxiety Inventory (STAI-T5) [35], and the Beck Hopelessness Scale (BHS-9) [36].

The MSPSS is a 12-item instrument that assesses one’s level of perceived social support from family, friends, and significant others. Responses to the 12 items are made on a 7-point scale, which ranges from 1 (very strongly disagree) to 7 (very strongly agree). An example item from the MSPSS is “I can talk about my problems with my friends.” In the original study that reported on the development of the MSPSS, Zimet and colleagues [33] reported satisfactory reliability coefficients of 0.88, 0.91, 0.87, and 0.85 for the overall scale, significant others subscale, family subscale, and friends subscale, respectively. A negative correlation between the MSPSS and both depression and anxiety provided evidence for the scale’s validity. The psychometric properties of the MSPSS have also been confirmed when used with South African youth [37].

The PSS Is a 10-item measure of the extent that participants perceive situations in their lives as stressful. It is scored on a 5-point scale that ranges from 0 (never) to 4 (very often). An example item from the PSS is “In the last month, how often have you felt that you were on top of things?” The author of the scale found that the PSS demonstrated satisfactory reliability in three different samples and the associations between perceived stress, life events, and depression served as evidence for its validity [2]. In South Africa, Makhubela [38] confirmed a bifactor structure for the PSS and also reported a Cronbach’s alpha of 0.79.

The CESD-10 is a shorter form of the 20-item CESD [39], which assesses depression. Responses to the 10 items are made on a 4-point scale ranging from 0 (rarely or none of the time) to 3 (most or all of the time). An example item of the CESD-10 is “I had trouble keeping my mind on what I was doing.” The original study that validated the 10-item version reported a reliability coefficient of 0.88 and demonstrated that the short form was as accurate as the original 20-item version in classifying participants with depressive symptoms. In South Africa, two different studies reported satisfactory reliability coefficients for the 20-item version of the CESD when used with a student sample [8] and a sample of teachers [40].

The STAI-T5 is a five-item short form of the 20-item trait scale of the State-Trait Anxiety Inventory [41], which assesses trait anxiety. Responses to the five items are made on a 4-point scale ranging from 1 (not at all) to 4 (very much so). An example item from the STAI-T5 is “I feel that difficulties are piling up so that I cannot overcome them.” Zsido and colleagues reported a reliability coefficient for the short version of the STAI-T of 0.86, and the correlation with the 20-item version was 0.88. One study in South Africa reported a reliability coefficient of 0.91 for the 20-item version of the scale [40].

The BHS-9 is a shorter form of the 20-item Beck Hopelessness Scale [42] and is a measure of the degree of pessimism about the future. The scoring format of the BHS-9 is dichotomous (yes/no), and an example item of the BHI-9 is “I never get what I want, so it’s foolish to want anything.” Balsamo and colleagues [36] used item response theory to develop the nine-item unidimensional version of the BHS and reported satisfactory reliability indices (alpha and Mokken scale reliability = 0.86).

### 2.3. Ethics

This study was conducted in accordance with the Guidelines of the Declaration of Helsinki, and ethical approval was obtained from the Humanities and Social Sciences Ethics Committee of the University of the Western Cape (ethics reference number: HS22/2/9, February 2022). There were no incentives provided for participation in the study, and participation in the study was voluntary and anonymous. The participants were given the opportunity to provide their informed consent on the landing page of the electronic link.

### 2.4. Analysis

All analyses were conducted using IBM SPSS for Windows version 28 (IBM Corp., Armonk, NY, USA). This included descriptive statistics (means and SDs), intercorrelations between variables (Pearson r), reliability levels (Cronbach’s alpha and McDonald’s omega), as well as mediation and moderation analyses.

The PROCESS macro for SPSS [43] was used for both the moderation (model 1) and mediation (model 4) analyses. In the mediation analysis, the significance of the indirect effect of perceived stress on indices of mental well-being was evaluated using 95% confidence intervals. In the moderation analysis, perceived stress and social support were mean-centered, and an interaction term was created using the mean-centered scores (perceived stress X social support). To examine the nature of any statistically significant interaction, the relationship between perceived stress and indices of mental well-being was plotted at different levels of social support (mean − 1 SD, mean, mean + 1 SD). For this purpose, the visualization code provided by the PROCESS macro was used.

## 3. Results

The descriptive statistics, coefficients of internal consistency (alpha) of variables, and the correlations between variables are reported in Table 2.

The reliability coefficients in Table 1 (α and ω) ranged between 0.84 and 0.93 and were regarded as satisfactory. There was a significant relationship between perceived stress and hopelessness (*r* = 0.47, *p* < 0.001), depression (*r* = 0.66, *p* < 0.001), and anxiety (*r* = 0.60, *p* < 0.001). For hopelessness, the effect size was medium, while for depression and anxiety, the effect size was large. The positive relationships indicate that high levels of perceived stress are associated with high levels of hopelessness, depression, and anxiety. All dimensions of social support were negatively related to the indices of mental health. In this regard, there was a significant negative relationship between support from significant others and hopelessness (*r* = −0.30, *p* < 0.001), depression (*r* = −0.35, *p* < 0.001), and anxiety (*r* = −0.22, *p* < 0.001). The observed correlations for hopelessness and depression indicate a medium-sized effect, while there was a small effect for anxiety. There was also a significant negative relationship between support from family and hopelessness (*r* = −0.33, *p* < 0.001), depression (*r* = −0.35, *p* < 0.001), and anxiety (*r* = −0.24, *p* < 0.001). The size of the coefficients indicated a medium-sized effect for hopelessness and depression and a small effect for anxiety. Similarly, there was a significant negative relationship between support from friends and hopelessness (*r* = −0.22, *p* < 0.001), depression (*r* = −0.32, *p* < 0.001), and anxiety (*r* = −0.21, *p* < 0.001). For hopelessness and anxiety, the observed correlation represented a small effect, while for depression, it was a medium-sized effect. The observed negative relationships would indicate that high levels of support from others are associated with low levels of hopelessness, depression, and anxiety.

We first examined the mediating role of the dimensions of social support in the relationship between perceived stress and indices of mental health. The results obtained with the PROCESS macro are presented in Table 3.

All of the direct effects of the dimensions of social support on depression and hopelessness were significant, except for the direct effects of support from friends on hopelessness. All indirect effects were also significant, indicating the mediating role of social support. In all instances, the indirect effects indicated that social support partially mediates the relationship between perceived stress and both depression and hopelessness, except for the inidrect effect of perceived stress on hopelessness via support from friends. In this instance, support from friends fully mediated the relationship between perceived stress and hopelessness, as the direct effect of support from friends on hopelessness was not significant when the mediator was present. There were no significant indirect effects of perceived stress on anxiety (i.e., the confidence interval contains zero), indicating that the social support dimensions did not play a mediating role in the relationship between perceived stress and anxiety.

**Figure 1:**
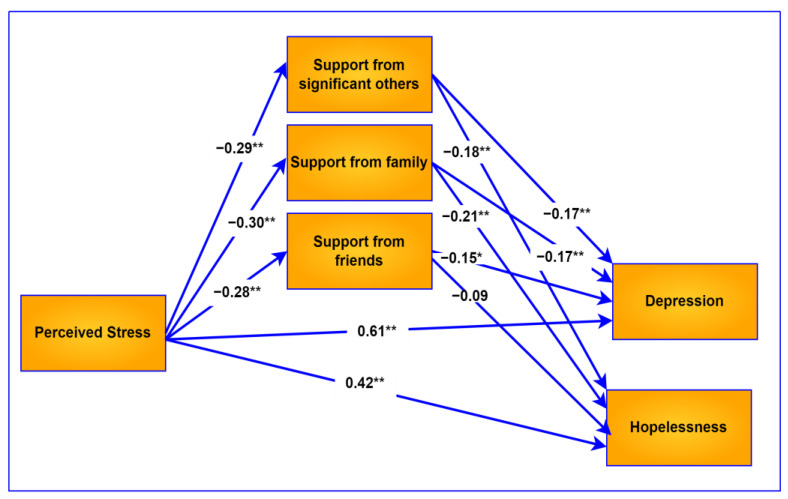
Conceptual model of the mediating role of social support. All regression coefficients are standardized. ** *p* < 0.001, * *p* < 0.01.

The mediation model for hopelessness and depression is visually presented in Figure 1. The coefficients in the model were obtained with the PROCESS macro, and the regression coefficients are standardized in the model.

Since the dimensions of social support did not mediate the relationship between perceived stress and anxiety, we investigated the potential moderating role of social support in the relationship between perceived stress and anxiety. The results of this moderation analysis are presented in Table 4.

**Figure 2:**
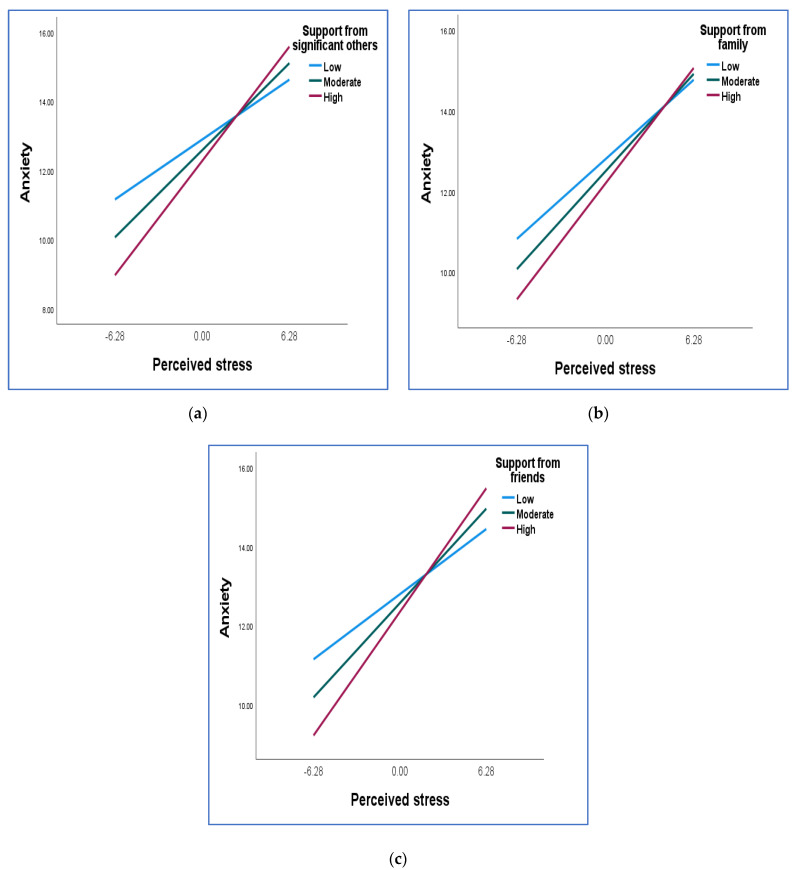
Illustration of the moderating role of the dimensions of social support in the relationship between perceived stress and anxiety: (**a**) the role of support from significant others; (**b**) the role of support from family; (**c**) the role of support from friends.

Table 4 indicates that the interaction term was significant for all three dimensions of support, indicating that support from significant others, family, and friends moderated the relationship between perceived stress and anxiety. The nature of this interaction is illustrated by the conditional effects of perceived stress at various levels of social support (mean − 1 SD, mean, mean + 1 SD) in Table 5, as well as the plot of the relationship between stress and anxiety for certain values of social support (mean − 1 SD, mean, mean + 1 SD) in Figure 2.

Table 5 indicates that while all coefficients at the various values of support from significant others were significant, the coefficient for 1 SD above the mean was the largest. This indicates that the relationship between perceived stress and anxiety was strongest for those who had a high level of support from their significant others. The same pattern was found for support from family and friends. The plots in Figure 2 illustrate the results in Table 5.

The three plots of the slopes of the regression line illustrate the moderating role of the dimensions of social support. At low levels of perceived stress, participants with high levels of support from significant others, family, and friends reported lower levels of anxiety than those with low levels of support. However, as levels of perceived stress increased, those with high levels of support reported higher levels of anxiety. Thus, the positive relationship between perceived stress and depression was stronger for those with high levels of support.

## 4. Discussion

The COVID-19 pandemic and the measures implemented for the prevention of its spread have differentially impacted university students’ mental health. The closure of campuses and the rapid transition to emergency remote online learning contributed to students’ heightened levels of fear and anxiety about their academic performance and career trajectories. Public health measures to reduce social gatherings such as mandatory social distancing, quarantine, and regulations led to a sense of disconnectedness among students and elevated levels of perceived stress [7]. Various studies of college students undertaken during the pandemic have reported increased levels of anxiety, depressive symptoms, insomnia, difficulty concentrating, heightened fear, and increased worries about academic performance for students [7,14]. Research has also highlighted the role of protective factors in mitigating adverse mental health outcomes [15]. The current study aimed to investigate the protective role of social support in the relationship between perceived stress and psychological distress among a sample of university students. There were several important findings.

First, the study confirmed that high levels of perceived stress are associated with high levels of hopelessness, depression, and anxiety. According to the transactional model of stress and coping [30], heightened primary appraisals of a life event as stressful lead to greater emotional distress. It is probable that the challenges that university students experienced due to pandemic prevention measures (e.g., social isolation, displacement from the academic environment, difficulties linked to online learning) contributed to heightened appraisals of stress and adverse mental health outcomes. Second, social support from family, friends, and significant others was associated with lower levels of hopelessness, depression, and anxiety, respectively. This can be explained by the buffering hypothesis in terms of which forms of social support can mitigate the impacts of adversity and reduce the risk of adverse mental health outcomes. For university students, knowing that they could rely on their family, friends, and significant others to manage stressors was likely beneficial in reducing distress associated with the pandemic. Research conducted during the pandemic [15,44] confirmed the social support’s protective role in reducing distress. Recent studies (e.g., [45]) have also found that students are inclined to share their difficulties and emotional struggles on social media platforms. Social networking sites such as Facebook, Instagram, and Twitter enable people to disclose personal experiences instantaneously and express their need for support. There is also evidence of a positive relationship between disclosure on social media platforms and actual support received [45]. In the context of the restrictions on social gatherings, students likely relied on social networking to meet their social support needs. Although the MSPSS does not include items pertaining to the functions of social support or providers of support, it is probable that this had a bearing on psychological outcomes. Social support can take many forms, including structural support (i.e., the size of the social network), functional support (i.e., meeting instrumental needs), emotional support, and informational support (i.e., the provision of information to assist with coping with difficult life events). These types of support can be provided by different sources, including family, friends, romantic partners, community organizations, peers, and the government. Furthermore, individuals operate within particular socio-cultural contexts, and their norms, beliefs, and values have a bearing on their willingness to access specific types of support. The effectiveness of social support in promoting mental health is dependent on the match between the source of support; the type of support provided; and the needs, developmental level, and life stage of the individual [46]. Existing research among university students and young adults has established that support from community networks (e.g., religious organizations, and youth centers), positive relationships with university staff, and support provided by the government (e.g., financial aid) are beneficial in promoting well-being [1,27]. In addition, emotional support, information support, and co-studying have been identified as salient resources for student mental health [47].

Third, in terms of the direct and mediating effects, social support was significant for depression and hopelessness but not for anxiety. This finding can be explained by social cognitive theory [48], according to which depression is associated with a past orientation and is usually caused by goal loss and the resulting feelings of sadness, rumination about the past, and disengagement from activities. In contrast, anxiety is associated with a future orientation. According to the uncertainty and anticipation model of anxiety [49], the experience of anxiety is grounded in uncertainty about future threats and the potential costs of these threats. It is associated with dysfunctional cognitive appraisals and behavioral responses to perceived stressors. When applied to the pandemic, uncertainty regarding the course of the outbreak and its longer-term impact on their lives, educational trajectory, and future careers could have sustained higher levels of anxiety among university students. Their anxiety may also have been aggravated by worries about contracting the virus and fears about not only their own health but also the health of their loved ones.

Finally, the study found that the relationship between perceived stress and depression was higher for those with high levels of social support. This was unexpected, as it implies that high levels of support amplified distress. Certain studies conducted during the COVID-19 outbreak have reported similar results. For example, in a study of Jordanian health care workers, Eman and colleagues [24] found that although these workers received high levels of support, they also experienced higher levels of psychological distress. For students, higher levels of support may lead to increased personal expectations of successful coping or reciprocation of support received from others. Fears of not meeting these expectations and not coping effectively may impact one’s mood and produce distress. Future research, possibly of a qualitative nature, may be beneficial toward further understanding this link.

The findings of the study have implications for interventions, confirming the protective role of social support in mitigating adverse mental health outcomes and highlighting the need for interventions to enhance social support resources among university students. Digital mental health interventions may be of particular benefit in promoting the well-being of students. Such interventions include social-media-based mental health tools that promote awareness of mental health problems, challenge the stigma associated with help-seeking, and encourage students to utilize available resources to manage emotional distress. University counseling centers can also provide psychoeducational workshops that teach practical strategies for identifying and accessing potential sources of social support, thereby reducing the incidence of mental health disorders [50].

This study has certain limitations. The cross-sectional design means that the hypothesized causal relationships need to be interpreted with caution, and longitudinal research is needed to further confirm the results. The study used self-report measures, and students with an interest in the topic may have been more likely to participate. In addition, social desirability bias may have impacted participants’ responses. The students participating in the study were enrolled at a single institution, and future research involving a more diverse sample and using different methodologies is recommended. A final limitation of the study is the focus on perceived individual support from friends, family, and significant others. Future studies that focus on different forms and sources of social support may be beneficial in elaborating on our findings.

## 5. Conclusions

University students are at risk of developing adverse mental health outcomes due to the distinct impact the pandemic has had on this population group. The current study confirmed that high levels of perceived stress are associated with greater psychological distress among students. Social support was instrumental in buffering against depression and hopelessness but not anxiety. This implies that in addition to enhancing social support resources, targeted interventions must be conducted to assist students in managing the uncertainty and anxiety associated with the pandemic. Furthermore, high levels of social support can potentially aggravate the association between perceived stress and depression. Intervention efforts, therefore, need to investigate students’ appraisals of support and the extent to which support is experienced as beneficial.

## Figures and Tables

**Table 1:** Description of the study sample (*n* = 322).

Variable	Categories	*n*	%
Gender	Women	248	77
	Men	68	21.1
	Transgender	2	0.6
	Non-binary	4	0.2
Area of residence	Urban	281	87.3
	Rural	41	12.7
Do you know people who have been infected with the coronavirus?	Yes	279	86.6
	No	30	9.3
	Don’t know	13	4.0
Have you ever tested positive for COVID-19?	Yes	82	25.5
	No	177	55.0
	Suspected COVID-19	63	19.6
Have you received a COVID-19 vaccine?	Yes	279	86.6
	No	43	13.4
Have you lost a family member due to COVID-19?	Yes	131	40.7
	No	191	59.3
Age	Mean = 26.01		SD = 10.19

**Table 2:** The descriptive statistics, as well as the reliability values of and correlations between variables.

Variable/Scale	1	2	3	4	5	6	7
1. Perceived stress							
2. Support from significant others	−0.29 **						
3. Support from family	−0.30 **	0.61 **					
4. Support from friends	−0.28 **	0.55 **	0.49 **				
5. Hopelessness	0.47 **	−0.30 **	−0.33 **	−0.22 **			
6. Depression	0.66 **	−0.35 **	−0.35 **	−0.32 **	0.50 **		
7. Anxiety	0.60 **	−0.22 **	−0.24	−0.21 **	0.46 **	0.66 **	
Mean	23.9	19.0	17.8	17.6	2.3	14.1	12.4
SD	6.3	7.3	6.9	7.0	2.4	6.8	4.1
Alpha	0.85	0.93	0.92	0.94	0.84	0.84	0.88
Omega	0.86	0.93	0.92	0.94	0.84	0.85	0.88

Note: ** *p* < 0.001.

**Table 3:** The direct and indirect effects of perceived stress and the dimensions of social support.

Effect	B	SE	95% CI	β	*p*
Direct effects					
Perceived stress -> depression	0.660	0.046	[0.569, 0.751]	0.612	<0.001
Perceived stress -> hopelessness	0.164	0.020	[0.126, 0.203]	0.422	<0.001
Perceived stress -> anxiety	0.389	0.031	[0.328, 0.449]	0.590	<0.001
Support from significant others -> depression	−0.157	0.040	[−0.235, −0.078]	−0.168	<0.001
Support from significant others -> hopelessness	−0.060	0.017	[−0.094, −0.027]	−0.180	<0.001
Support from significant others -> anxiety	−0.028	0.026	[−0.080, 0.024]	−0.049	0.294
Support from family -> depression	−0.161	0.042	[−0.244, −0.078	−0.165	<0.001
Support from family -> hopelessness	−0.073	0.018	[−0.108, −0.038]	−0.207	<0.001
Support from family -> anxiety	−0.039	0.028	[−0.094, 0.015]	−0.066	0.159
Support from friends -> depression	−0.145	0.042	[−0.227, −0.064]	0.151	<0.001
Support from friends -> hopelessness	−0.033	0.018	[−0.068, 0.002]	−0.093	0.068
Support from friends -> anxiety	−0.024	0.027	[−0.078, 0.030]	−0.040	0.387
Indirect effects					
Perceived stress -> support from significant others -> depression	0.053	0.017	[0.023, 0.091]	0.049	—
Perceived stress -> support from family -> depression	0.053	0.018	[0.020, 0.093]	0.050	—
Perceived stress -> support from friends -> depression	0.046	0.017	[0.015, 0.082]	0.042	—
Perceived stress -> support from significant others -> hopelessness	0.020	0.007	[0.008, 0.036]	0.052	—
Perceived stress -> support from family -> hopelessness	0.024	0.008	[0.011, 0.041]	0.062	—
Perceived stress-> support from friends -> hopelessness	0.010	0.006	[0.000, 0.022]	0.026	—
Perceived stress -> support from significant others -> anxiety	0.009	0.010	[−0.010, 0.029]	0.014	
Perceived stress -> support from family -> anxiety	0.013	0.010	[−0.006, 0.035]	0.020	
Perceived stress -> support from friends -> anxiety	0.007	0.009	[−0.010, 0.027]	0.011	

Note. B = unstandardized coefficient; SE = standard error; CI = confidence interval; β = standardized coefficient.

**Table 4:** The moderating role of dimensions of social support.

Variable	B	SE	95% CI	*p*
Anxiety as outcome variable				
Perceived Stress	0.402	0.030	[0.343, 0.460]	<0.001
Support from significant others	−0.042	0.026	[−0.093, 0.008]	0.100
Support from family	−0.044	0.028	[−0.098, 0.011]	0.116
Support from friends	−0.032	0.027	[−0.085, 0.021]	0.235
Perceived stress X support from significant others	0.017	0.004	[0.010, 0.024]	<0.001
Perceived stress X support from family	0.010	0.004	[0.002, 0.018]	0.012
Perceived stress X support from friends	0.017	0.004	[0.008, 0.025]	<0.001

Note: B = unstandardized coefficient; SE = standard error; CI = confidence interval.

**Table 5:** Conditional effects of perceived stress on anxiety at the −1 SD, mean, and + 1 SD level of social support.

Values of Social Support	Effect	SE	95% CI	*p*
Support from significant others				
Low (mean − 1 SD)	0.276	0.038	[0.201,0.351]	<0.001
Moderate (mean)	0.402	0.030	[0.343, 0.460]	<0.001
High (mean + 1 SD)	0.527	0.042	[0.445, 0.609]	<0.001
Support from family				
Low (mean − 1 SD)	0.315	0.041	[0.234, 0.396]	<0.001
Moderate (mean)	0.386	0.030	[0.326, 0.446]	<0.001
High (mean + 1 SD)	0.458	0.042	[0.375, 0.540]	<0.001
Support from friends				
Low (mean − 1 SD)	0.263	0.045	[0.175, 0.351]	<0.001
Moderate (mean)	0.381	0.030	[0.322, 0.440]	<0.001
High (mean + 1 SD)	0.499	0.041	[0.418, 0.580]	<0.001

Note: SE = standard error; CI = confidence interval.

## Data Availability

The raw data supporting the conclusions of this article will be made available by the authors, without undue reservation.

## References

[1] Szkody E., Stearns M., Stanhope L., McKinney C. (2021). Stress-Buffering Role of Social Support during COVID-19. Fam. Process.

[2] Cohen S., Spacapan S., Oskamp S. (1988). Perceived stress in a probability sample of the United States. The Social Psychology of Health.

[3] Yan L., Gan Y., Ding X., Wu J., Duan H. (2021). The relationship between perceived stress and emotional distress during the COVID-19 outbreak: Effects of boredom proneness and coping style. J. Anxiety Disord..

[4] Robinson E., Sutin A.R., Daly M., Jones A. (2022). A systematic review and meta-analysis of longitudinal cohort studies comparing mental health before versus during the COVID-19 pandemic in 2020. J. Affect. Disord..

[5] Wu T., Jia X., Shi H., Niu J., Yin X., Xie J., Wang X. (2021). Prevalence of mental health problems during the COVID-19 pandemic: A systematic review and meta-analysis. J. Affect. Disord..

[6] Magnúsdóttir I., Lovik A., Unnarsdóttir A.B., McCartney D., Ask H., Kõiv K., Christoffersen L.A.N., Johnson S.U., Hauksdóttir A., Fawns-Ritchie C. (2022). Acute COVID-19 severity and mental health morbidity trajectories in patient populations of six nations: An observational study. Lancet Public Health.

[7] Chen T., Lucock M. (2022). The mental health of university students during the COVID-19 pandemic: An online survey in the UK. PLoS ONE.

[8] Padmanabhanunni A., Pretorius T. (2022). Behaviour is the key in a pandemic: The direct and indirect effects of COVID-19-related variables on psychological wellbeing. Psychol. Rep..

[9] Elharake J.A., Akbar F., Malik A.A., Gilliam W., Omer S.B. (2022). Mental Health Impact of COVID-19 among Children and College Students: A Systematic Review. Child Psychiatry Hum. Dev..

[10] Torales J., Ríos-González C., Barrios I., O’Higgins M., González I., García O., Castaldelli-Maia J.M., Ventriglio A. (2020). Self-Perceived Stress During the Quarantine of COVID-19 Pandemic in Paraguay: An Exploratory Survey. Front. Psychiatry.

[11] Valikhani A., Kashani V.O., Rahmanian M., Sattarian R., Rahmati Kankat L., Mills P.J. (2020). Examining the mediating role of perceived stress in the relationship between mindfulness and quality of life and mental health: Testing the mindfulness stress buffering model. Anxiety Stress Coping.

[12] Stamatis C.A., Broos H.C., Hudiburgh S.E., Dale S.K., Timpano K.R. (2022). A longitudinal investigation of COVID-19 pandemic experiences and mental health among university students. Br. J. Clin. Psychol..

[13] Chaudhary A.P., Sonar N.S., Tr J., Banerjee M., Yadav S. (2021). Impact of the COVID-19 Pandemic on the Mental Health of College Students in India: Cross-sectional Web-Based Study. JMIRx Med.

[14] Faisal R.A., Jobe M.C., Ahmed O., Sharker T. (2022). Mental Health Status, Anxiety, and Depression Levels of Bangladeshi University Students During the COVID-19 Pandemic. Int. J. Ment. Health Addict..

[15] Grey I., Arora T., Thomas J., Saneh A., Tohme P., Abi-Habib R. (2020). The role of perceived social support on depression and sleep during the COVID-19 pandemic. Psychiatry Res..

[16] Riepenhausen A., Veer I.M., Wackerhagen C., Reppmann Z.C., Köber G., Ayuso-Mateos J.L., Bögemann S.A., Corrao G., Felez-Nobrega M., Abad J.M.H. (2022). Coping with COVID: Risk and resilience factors for mental health in a German representative panel study. Psychol. Med..

[17] Chew Q.H., Wei K.C., Vasoo S., Chua H.C., Sim K. (2020). Narrative synthesis of psychological and coping responses towards emerging infectious disease outbreaks in the general population: Practical considerations for the COVID-19 pandemic. Singap. Med. J..

[18] Song E., Yoo H.J. (2020). Impact of Social Support and Social Trust on Public Viral Risk Response: A COVID-19 Survey Study. Int. J. Environ. Res. Public Health.

[19] Li F., Luo S., Mu W., Li Y., Ye L., Zheng X., Xu B., Ding Y., Ling P., Zhou M. (2021). Effects of sources of social support and resilience on the mental health of different age groups during the COVID-19 pandemic. BMC Psychiatry.

[20] Wang J., Mann F., Lloyd-Evans B., Ma R., Johnson S. (2018). Associations between loneliness and perceived social support and outcomes of mental health problems: A systematic review. BMC Psychiatry.

[21] Okabayashi H., Liang J., Krause N., Akiyama H., Sugisawa H. (2004). Mental health among older adults in Japan: Do sources of social support and negative interaction make a difference?. Soc. Sci. Med..

[22] Alsubaie M.M., Stain H.J., Webster L.A.D., Wadman R. (2019). The role of sources of social support on depression and quality of life for university students. Int. J. Adolesc. Youth.

[23] Qi M., Zhou S.-J., Guo Z.-C., Zhang L.-G., Min H.-J., Li X.-M., Chen J.-X. (2020). The Effect of Social Support on Mental Health in Chinese Adolescents During the Outbreak of COVID-19. J. Adolesc. Health.

[24] Alnazly E., Khraisat O.M., Al-Bashaireh A.M., Bryant C.L. (2021). Anxiety, depression, stress, fear and social support during COVID-19 pandemic among Jordanian healthcare workers. PLoS ONE.

[25] Labrague L.J. (2021). Psychological resilience, coping behaviours and social support among health care workers during the COVID-19 pandemic: A systematic review of quantitative studies. J. Nurs. Manag..

[26] Ifdil I., Fadli R.P., Suranata K., Zola N., Ardi Z. (2020). Online mental health services in Indonesia during the COVID-19 outbreak. Asian J. Psychiatry.

[27] Shah A.U.M., Safri S.N.A., Thevadas R., Noordin N.K., Rahman A.A., Sekawi Z., Ideris A., Sultan M.T.H. (2020). COVID-19 outbreak in Malaysia: Actions taken by the Malaysian government. Int. J. Infect. Dis..

[28] Kennelly B., O’Callaghan M., Coughlan D., Cullinan J., Doherty E., Glynn L., Moloney E., Queally M. (2020). The COVID-19 pandemic in Ireland: An overview of the health service and economic policy response. Health Policy Technol..

[29] Schulder T., Rudenstine S., Bhatt K.J., McNeal K., Ettman C.K., Galea S. (2022). A multilevel approach to social support as a determinant of mental health during COVID-19. J. Community Psychol..

[30] Lazarus R.S., Folkman S. (1984). Stress, Appraisal, and Coping.

[31] Cohen S., Wills T.A. (1985). Stress, Social Support, and the Buffering Hypothesis. Psychol. Bull..

[32] Bekiros S., Jahanshahi H., Munoz-Pacheco J.M. (2022). A new buffering theory of social support and psychological stress. PLoS ONE.

[33] Zimet G.D., Dahlem N.W., Zimet S.G., Farley G.K. (1988). The Multidimensional Scale of Perceived Social Support. J. Personal. Assess..

[34] Zhang W., O’Brien N., Forrest J.I., Salters K.A., Patterson T.L., Montaner J.S.G., Hogg R.S., Lima V.D. (2012). Validating a shortened depression scale (10 item CES-D) among HIV-positive people in British Columbia, Canada. PLoS ONE.

[35] Zsido A.N., Teleki S.A., Csokasi K., Rozsa S., Bandi S.A. (2020). Development of the short version of the spielberger state—Trait anxiety inventory. Psychiatry Res..

[36] Balsamo M., Carlucci L., Innamorati M., Lester D., Pompili M. (2020). Further Insights Into the Beck Hopelessness Scale (BHS): Unidimensionality Among Psychiatric Inpatients. Front. Psychiatry.

[37] Bruwer B., Emsley R., Kidd M., Lochner C., Seedat S. (2008). Psychometric properties of the Multidimensional Scale of Perceived Social Support in youth. Compr. Psychiatry.

[38] Makhubela M. (2022). Assessing psychological stress in South African university students: Measurement validity of the perceived stress scale (PSS-10) in diverse populations. Curr. Psychol..

[39] Radloff L.S. (1977). The CES-D scale: A self-report depression scale for research in the general population. Appl. Psychol. Meas..

[40] Pretorius T.B., Padmanabhanunni A. (2022). Validation of the Connor-Davidson Resilience Scale-10 in South Africa: Item Response Theory and Classical Test Theory. Psychol. Res. Behav. Manag..

[41] Spielberger C.D. (1983). Manual for the State-Trait Anxiety Inventory.

[42] Beck A.T., Weissman A., Lester D., Trexler L. (1974). The measurement of pessimism: The hopelessness scale. J. Consult. Clin. Psychol..

[43] Hayes A.F. (2017). Introduction to Mediation, Moderation, and Conditional Process Analysis: A Regression-Based Approach.

[44] Ghafari R., Mirghafourvand M., Rouhi M., Osouli Tabrizi S. (2021). Mental health and its relationship with social support in Iranian students during the COVID-19 pandemic. BMC Psychol..

[45] Zhang R. (2017). The stress-buffering effect of self-disclosure on Facebook: An examination of stressful life events, social support, and mental health among college students. Comput. Hum. Behav..

[46] Lauren M.S., Robert H.P., Dennis S.C., Linda C.M., Steven M.S. (2015). How does social support enhance resilience in the trauma-exposed individual?. Ecol. Soc..

[47] Elmer T., Mepham K., Stadtfeld C. (2020). Students under lockdown: Comparisons of students’ social networks and mental health before and during the COVID-19 crisis in Switzerland. PLoS ONE.

[48] Beck A.T., Haigh E.A.P. (2014). Advances in Cognitive Theory and Therapy: The Generic Cognitive Model. Annu. Rev. Clin. Psychol..

[49] Grupe D.W., Nitschke J.B. (2013). Uncertainty and anticipation in anxiety: An integrated neurobiological and psychological perspective. Nat. Rev. Neurosci..

[50] Harandi T.F., Taghinasab M.M., Nayeri T.D. (2017). The correlation of social support with mental health: A meta-analysis. Electron. Physician.

